# Prospective Alternate Hosts of an Emerging Polerovirus in Cotton Landscapes in the Southeastern United States

**DOI:** 10.3390/v14102249

**Published:** 2022-10-13

**Authors:** Sudeep Pandey, Sudeep Bag, Phillip Roberts, Kassie Conner, Kipling S. Balkcom, Andrew J. Price, Alana L. Jacobson, Rajagopalbabu Srinivasan

**Affiliations:** 1 Department of Entomology, University of Georgia, 1109 Experiment Street, Griffin, GA 30223, USA; 2 Department of Plant Pathology, University of Georgia, 2360 Rainwater Road, Tifton, GA 31793, USA; 3 Department of Entomology, University of Georgia, 2360 Rainwater Road, Tifton, GA 31793, USA; 4 Alabama Cooperative Extension System, Auburn University, 961 S. Donahue Dr., Auburn, AL 36849, USA; 5 USDA, ARS, Soil Dynamics Research, 411 S. Donahue Dr., Auburn, AL 36832, USA; 6 Department of Entomology and Plant Pathology, Auburn University, 310 Funchess Hall, Auburn, AL 36849, USA

**Keywords:** CLRDV, *Aphis gossypii*, virus transmission, inoculum sources, vector reservoirs

## Abstract

The identification of alternate hosts that can act as virus inoculum sources and vector reservoirs in the landscape is critical to understanding virus epidemics. Cotton leafroll dwarf virus (CLRDV) is a serious pathogen in cotton production and is transmitted by the cotton/melon aphid, *Aphis gossypii*, in a persistent, circulative, and non-propagative manner. CLRDV was first reported in the United States in Alabama in 2017, and thereafter in several cotton-producing states. CLRDV has since established itself in the southeastern United States. The role of alternate hosts in CLRDV establishment is not clear. Fourteen common plant species in the landscape, including crops, weeds, and ornamentals (cotton, hollyhock, marshmallow, country mallow, abutilon, arrowleaf sida, okra, hibiscus, squash, chickpea, evening primrose, henbit, Palmer amaranth, and prickly sida) were tested as potential alternate hosts of CLRDV along with an experimental host (*Nicotiana benthamiana*) via aphid-mediated transmission assays. CLRDV was detected following inoculation in hibiscus, okra, *N. benthamiana*, Palmer amaranth, and prickly sida by RT-PCR, but not in the others. CLRDV accumulation determined by RT-qPCR was the highest in *N. benthamiana* compared with cotton and other hosts. However, aphids feeding on CLRDV-infected prickly sida, hibiscus, and okra alone were able to acquire CLRDV and back-transmit it to non-infected cotton seedlings. Additionally, some of the alternate CLRDV hosts supported aphid development on par with cotton. However, in a few instances, aphid fitness was reduced when compared with cotton. Overall, this study demonstrated that plant hosts in the agricultural landscape can serve as CLRDV inoculum sources and as aphid reservoirs and could possibly play a role in the reoccurring epidemics of CLRDV in the southeastern United States.

## 1. Introduction

Plant viruses infecting annual agricultural crops in sub-tropical and temperate regions can also infect alternate hosts in proximity, which in turn act as a feedback mechanism for maintaining the virus in the landscape [1]. Alternate hosts could include other crop species as well as ornamentals and weeds that persist in the landscape even during crop-free instances. To influence epidemics of vector-transmitted persistent viruses, alternate hosts must serve as vector reservoirs and as virus inoculum sources [2]. Cotton leafroll dwarf virus (CLRDV) is a relatively new introduction to the southeastern United States, where cotton (*Gossypium hirsutum* L.) is grown in nearly two million acres, and high incidence of CLRDV has raised serious production concerns [3,4]. However, the various factors that influence its annual epidemics remain uncharacterized. CLRDV is the causative organism of Cotton Blue Disease (CBD), previously reported in Africa, South America, and Asia [5,6,7]. The virus was first reported in the United States in Alabama in 2017 [8]. Subsequently, the virus has been reported in several cotton-producing states, i.e., Georgia, Texas, Mississippi, Louisiana, South Carolina, North Carolina, Florida, Louisiana, Arkansas, and Kansas [9,10,11,12,13,14,15,16,17,18]. Typical symptoms associated with cotton leafroll dwarf virus in South America are stunting, leaf rolling, vein yellowing, dark-green leaves, small bolls, and yield losses of up to 80% [19,20]. Infection of cotton plants by the United States strain of CLRDV produces a different suite of symptoms when compared with cotton blue disease symptoms, and hence the phenotype is called as the cotton leafroll dwarf disease (CLRDD) in the United States [3]. A recent study from Georgia, United States, has reported that negative impacts of CLRDD on physiological processes of cotton plants can at times result in complete yield loss [21].

Cotton leafroll dwarf virus is a species in the genus *Polerovirus* and belongs to the family *Solemoviridae* [22]. CLRDV is a phloem-limited, positive-sense, single-stranded RNA virus with seven open reading frames (ORFs) grouped into two blocks and separated by a non-coding region. The CLRDV genome is 5.8 kb long. Three ORFs (ORF0, ORF1, and ORF2) are located at the 5′ end, whereas four ORFs (ORF3a, ORF3, ORF4, and ORF5) are located at the 3′ region. The ORF0 encodes for the P0 protein that functions as an RNA silencing suppressor; overlapping ORFs 1 and 2 encode for two virus replication related proteins (P1, P1–P2). Similarly, ORF3a encodes for long distance movement protein (P3a) associated with systemic movement of the virus within the host, ORF3 encodes for the coat protein (P3), ORF4 encodes for the movement protein (P4) associated with cell-to-cell movement, and ORF5 encodes for the P5 protein (read-through domain). Proteins P3–P5 have been associated with aphid-transmission and virus accumulation in plants [6,23,24,25,26].

CLRDV is transmitted by the cotton/melon aphid (*Aphis gossypii* Glover), which is an annual pest in the southeastern United States cotton production region [27,28,29]. *Aphis gossypii* transmits CLRDV to cotton plants in a circulative and non-propagative manner [29]. Virus ingestion occurs when aphids feed on the phloem of virus-infected plants, once in the midgut, the virus traverses into the hemocoel via endocytosis, and from there it endocytoses into the accessory salivary glands [30,31]. Both winged and wingless morphs of *A. gossypii* can transmit the virus after a 48 h inoculation access period for up to 12 days [32,33]. CLRDV also was detected in the soybean aphid (*Aphis glycines* M.) via next-generation sequencing; however, its ability to transmit CLRDV is not known [34]. None of the other aphid species are reported to transmit CLRDV to cotton.

Cotton is grown as a spring/summer crop in the southeastern United States lasting from April to October. Cotton fields in the landscape are typically surrounded by other crops, weeds, and ornamentals. CLRDV has been detected annually since its introduction suggesting that it has established in the region and survives cotton-free periods on alternate hosts. A recent field survey in Georgia reported CLRDV from 23 weed species belonging to 16 families, overwintering cotton stalks, and regrowth leaves [35]. The role of alternate hosts in the Southeast on CLRDV epidemics is not completely understood. Specifically, their role as virus inoculum sources and vector reservoirs through aphid-mediated transmission has not been established.

This study evaluated different plant species as alternate hosts of CLRDV and the vector under greenhouse conditions. The objectives of this study were to: (i) assess the susceptibility of fourteen plant species to CLRDV via aphid-mediated inoculation, (ii) quantitate CLRDV accumulation in the resulting CLRDV-infected hosts, (iii) evaluate the ability of aphids to acquire CLRDV from alternate hosts, (iv) determine whether aphids can back-transmit CLRDV to cotton from infected alternate hosts, and (v) assess the fitness of *A. gossypii* on CLRDV alternate hosts.

## 2. Materials and Methods

### 2.1. Maintenance of Plants, Virus Isolate, and the Insect Colony

CLRDV-infected cotton plants were collected from a cotton field at the University of Georgia, Tifton campus, GA, USA, in September 2020. CLRDV was subsequently maintained in cotton plants by repeated aphid-mediated inoculation of two-true leaf stage cotton (*G. hirsutum* L. cv. PHY 339 WRF) plants in the greenhouse with approximately 100 aphids per plant. Inoculated plants were placed in aphid-proof cages of size 47.5 (l) × 47.5 (w) × 93 (h) cm^3^ (Megaview Science Co., Taichung, Taiwan) in the greenhouse at 25 °C, 60% relative humidity, and 14 h L:10 h D photoperiod. Non-viruliferous cotton aphids were first collected from a cotton field at the University of Georgia, Tifton Campus, in 2017, and subsequently maintained on non-infected cotton plants under greenhouse conditions stated above.

Plant/weed species commonly present in southeastern United States were selected for this experiment. Cotton seeds (*G. hirsutum* L. cv. PHY 339 WRF) were obtained from the University of Georgia extension services, Tifton, GA, USA. Seeds of fourteen other plant species used for this experiment also were either field-collected or commercially obtained: prickly sida *(Sida spinosa* L.) from Azlin Seed Service (Leland, MS, USA); Abutilon Hybridum Bellvue Mix (*Abutilon* spp. Mill), marshmallow (*Malva parviflora* L.), and hollyhock (*Alcea rosea* L.) from Outsidepride (Independence, OR, USA); hibiscus (*Hibiscus acetosella* Welw. Ex Hiern.) from Johnny’s Selected Seeds (Winslow, ME, USA); summer squash (*Cucurbita pepo* L.) from Holmes Seed Company (Canton, OH, USA)*;* country mallow (*Sida cordifolia* L.) from Asklepios (Bad Liebenzell, Germany); chickpea (*Cicer arietinum* L.) from Country Creek LLC Brand (Brentwood, MO, USA); and okra (*Abelmoschus esculentus* L. cv. ‘’Clemson Spineless 80’) from the University of Georgia extension services. *Nicotiana benthamiana* Domin seeds were kindly provided by Dr. Scott Adkins, USDA-ARS, Fort Pierce, FL, USA. Arrowleaf sida (*Sida rhombifolia* L.) and Palmer amaranth (*Palmer amaranth Palmeri* S.Wats.) seeds were collected at the University of Georgia Tifton Campus. Evening primrose (*Oenothera laciniata* L.) and henbit (*Lamium amplexiculae* L.) seeds were collected from the University of Georgia Griffin campus, GA, USA. All plants were grown in Sunshine propagation mix (SunGro Horticulture Industries, Bellevue, WA, USA) in 5 cm diameter plastic pots (depth 4 cm). Two to four seeds of each host were planted per pot and housed in insect-proof cages. One to two weeks post-germination, seedlings were thinned to one per pot. Plants were fertilized weekly with water-soluble Miracle-Gro (Scotts Miracle-Gro products, Inc., Marysville, OH, USA) at 0.5 g/L and watered approximately twice a week. Plants at the two-true-leaf stage (three-to-five weeks old) were used for experiments.

### 2.2. Transmission of CLRDV to Test Plants

Three to five weeks post-germination, ten potted plants of individual plant species were placed in a new insect-proof cage. Approximately 100 adult viruliferous aphids obtained after 72 h of acquisition access period (AAP) on CLRDV-infected cotton plants were caged on the abaxial side of each non-infected plant using leaf cages (Table S1). Aphids were provided with a 72 h inoculation access period (IAP) on test plants and leaf cages were removed. After the IAP, plants were sprayed with imidacloprid (1% Montana 2F, Rotam, Greensboro, NC, USA) to eliminate aphids. The topmost young leaves with petiole were collected from each inoculated test plant at three weeks post-inoculation, tested for the presence of CLRDV by RT-PCR, and quantitated via RT-qPCR. The experiment was conducted three times for each species (n = 28–30 for each species).

### 2.3. Detection and Quantitation of CLRDV

For CLRDV detection, total RNA was extracted from leaf samples of test plants using Spectrum^TM^ Plant Total RNA kit (Sigma-Aldrich, St. Louis, MO, USA) following manufacturer’s instructions. Complementary DNA (cDNA) was synthesized using 1 μg total RNA and Go-Script reverse transcription system (Promega Corporation, Madison, WI, USA) according to manufacturer’s instructions. Random hexamers were used for cDNA synthesis, and cDNA from each sample was used as a template for RT-PCR and RT-qPCR using specific primer pairs (Table 1).

For PCR, 5 μL of GoTaq Green Mastermix buffer (2X) (Promega, Madison, WI, USA) was combined with 0.5 μL each of forward and reverse primers (0.3 μM) (Table 1), 2 μL of cDNA, and sterile nuclease-free water for a final volume of 10 μL. PCR was performed using a T-100 thermocycler (Bio-Rad, Hercules, CA, USA). Conditions of the PCR reaction were an initial denaturation step of three minutes at 94 °C, followed by 40 cycles of denaturation at 94 °C for 30 s, annealing at 62 °C for 20 s and 56 °C for 10 s, and extension at 72 °C for one minute. A final extension step at 72 °C for three minutes was included [5]. Polymerase chain reaction amplified cDNA fragments were analyzed by agarose gel (1%) electrophoresis in 1× Tris-acetate (TAE) buffer (40 mM Trisacetate and 2 mM ethylenediamine tetraacetic acid; pH, 8.0), stained with gel red, and visualized at 302 nm using the MultiDoc-It Imaging System (UVP, Jena, Germany).

Virus accumulation in infected hosts was estimated through RT-qPCR using primer pairs CLRDV-CP5L and CLRDV-CP5R and a CLRDV-CP5 probe (Table 1). Primers were designed to partially amplify the coat protein region of the virus using the Primer3 (v 0.4.0) software (Cambridge, MA, USA). Quantitative PCR was performed using 10 μL GoTaq^®^ probe qPCR Master Mix (Promega, Madison, WI, USA) mixed with primers (0.3 μM) and the probe (0.5 μL each), 5 μL of cDNA, and nuclease-free distilled water for a final volume of 20 μL in a QuantStudio™ 3 Real-Time PCR System (Applied Biosystems by Thermo Fisher Scientific, Waltham, MA, USA). The qPCR conditions included an initial denaturation step at 95 °C for three minutes followed by 40 cycles at 95 °C for 15 s and 60 °C for one minute. Each sample was technically duplicated. The absolute number of copies present in each sample was quantitated in reference to a standard curve generated with a series of eight 10-fold dilutions of CLRDV_pJET1.2 plasmids containing partial sequences of the ORF3 fragment (GenScript, NJ, USA) [36].

### 2.4. Virus Acquisition from Alternate Hosts by Aphids and CLRDV Quantitation

The ability of *A. gossypii* to acquire CLRDV from infected cotton and other hosts (that tested positive for CLRDV) was assessed by confining non-viruliferous aphids on leaves at upper one-third of the infected hosts using leaf cages. An AAP of 72 h was provided for aphids and then transferred to CLRDV non-host, summer squash, for an additional 72 h to facilitate gut clearing. Each sample consisting of a pool of five insects were collected and total RNA was extracted using Qiagen RNA mini-Kit (Valencia, CA, USA) as per the manufacturer’s instructions. Detection of CLRDV by RT-PCR and quantitation of virus by RT-qPCR were carried out as per the protocols described above for plants. Three replications were included in each experiment and the experiment was repeated five times more. Approximately, 90 aphids (pooled in 3 × 6 samples) were processed from each plant host.

### 2.5. Back-Transmission Assays to Cotton

Hosts that tested positive for CLRDV were used as inoculum sources in the CLRDV back-transmission assays with non-infected cotton plants as recipients. Non-viruliferous aphids (~100) were caged to CLRDV-infected alternate hosts and provided with an AAP of 72 h. Then, the aphids were caged on to three-week-old (two-true leaf stage) non-infected cotton plants and provided with an IAP of 72 h. After the IAP, the plants were sprayed with imidacloprid (1% Montana 2F, Rotam, Greensboro, NC, USA) to eliminate aphids and placed in insect-proof cages for three weeks. Leaf samples were collected from the cotton plants and used further for virus detection and quantitation as previously described. The experiment was conducted with 10 cotton plants for each alternate host species and repeated two times (n = 30 cotton plants per alternate host species).

### 2.6. Alternate Plant Species as Hosts of Aphids

The alternate hosts of CLRDV identified from the above experiments were tested for their suitability as hosts of *A. gossypii*. One adult aphid from the non-viruliferous colony maintained on non-infected cotton plants was caged on the abaxial surface of the leaf of test plants. Adults were monitored every 24 h. When offspring were observed, only one nymph was left in the leaf cage, and the remaining adult and nymphs were removed. A single nymph per cage was monitored throughout the life cycle. Fitness parameters such as aphid survival (nymph to adult), nymphal period, adult period (pre-reproductive, reproductive, and post-reproductive), total fecundity, and intrinsic rate of increase were evaluated [37,38]. The total number of nymphs laid by an adult in the subsequent generation was recorded and removed from the cage. Nymphal period refers to duration in days from the first day of larviposition to the first day of adulthood, whereas the adult period was the duration in days from the first day of adulthood to death. The intrinsic rate of increase for each aphid was calculated using the following equation [39]:

$$r_{m}=0.747\frac{log_{e}N_{d}}{d}$$

where *N_d_* = number of nymphs produced during reproductive period, *d* = pre-reproductive period in days.

The experiment was conducted using one plant per host with ten leaf cages attached to each plant. Similar size leaves from the upper one-third to middle portion of the plant were selected. The experiment was repeated twice more. Therefore, 30 leaf cages on three plants were monitored per host.

### 2.7. Statistical Analyses

Data from all experimental repeats were pooled for statistical analysis. CLRDV infection in plants and aphids were analyzed assuming a binomial response (infected vs. noninfected) using the agricolae package in R version 4.1.0 using a generalized linear mixed model [40]. Treatments were considered as fixed effects and the experiment replications and repeats were considered as random effects. Data for CLRDV accumulation in plants and aphids were analyzed using the agricolae package after log transformation using a linear mixed model. Means for virus accumulation were compared with Tukey-HSD post hoc test. Statistical differences were considered significant at *p* < 0.05. Aphid survival was analyzed assuming a binomial response (live vs. dead), also using the agricolae package. Total fecundity and intrinsic rate of increase were analyzed using a linear mixed model with treatments as fixed effects and replications as random effects as described above. Aphid developmental time parameters such as nymphal period, pre-reproductive period, reproductive period, post-reproductive period, adult period, and total life span were analyzed using a non-parametric Kruskal–Wallis test using the agricolae package.

## 3. Results

### 3.1. CLRDV Host Range and Virus Quantitation

**Figure 1:**
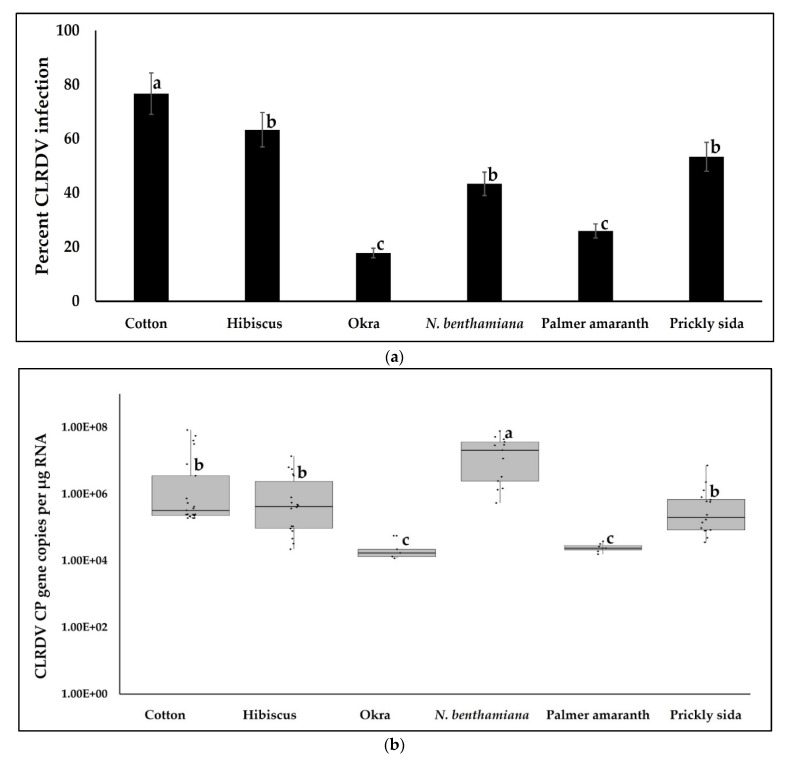
CLRDV infection in hosts. (**a**) Bars with standard errors represent mean percentages of infected hosts after caging 100 viruliferous aphids on the abaxial surface of a two-true leaf stage plant after a 72 h of IAP. Inoculated plants were tested three weeks post-inoculation by RT-PCR. (**b**) Boxes with whiskers represent CLRDV accumulation on infected hosts that tested positive for CLRDV three weeks after aphid-mediated inoculation. The CLRDV CP copies were estimated by RT-qPCR absolute quantitation using plasmids containing CLRDV CP gene inserts as standards. Y-axis is represented on a logarithmic scale. Different letters on bars and boxes indicate significant differences between treatments.

Aphid-mediated CLRDV inoculation to cotton and fourteen plant species using leaf cages resulted in CLRDV infection of cotton and five other plant species, i.e., hibiscus, okra, *N. benthamiana*, Palmer amaranth, and prickly sida (Figure 1a). The percentage of CLRDV-infected plants was significantly different among hosts (χ^2^ = 25.867, df = 5, 172, *p* < 0.0001). CLRDV infection was the highest in cotton and lowest in Palmer amaranth and okra. CLRDV infection percentages in hibiscus, prickly sida, and *N. benthamiana* did not vary from each other (Figure 1a). Typical CLRDV-associated symptoms such as reddening, downward curling, internodal shortening, and stunting were not observed in any of the infected hosts including cotton (Figure S1). None of the plants subjected to inoculation by non-viruliferous aphids tested positive for CLRDV by RT-PCR (Table S2).

Hosts that tested positive for CLRDV were used to assess virus accumulation by RT-qPCR. CLRDV CP copies in hosts varied significantly (*F* = 15.9976, df = 5, 72, *p* < 0.0001). CLRDV CP copies were the highest in *N. benthamiana* and the lowest in Palmer amaranth and okra. CLRDV CP copies in cotton, hibiscus, and prickly sida did not differ from each other (Figure 1b). RT-qPCR results also indicated that plants subjected to inoculation by non-viruliferous aphids did not accumulate any CLRDV gene copies.

### 3.2. CLRDV Acquisition by Aphids from Cotton and Alternate Hosts and Virus Quantitation

**Figure 2:**
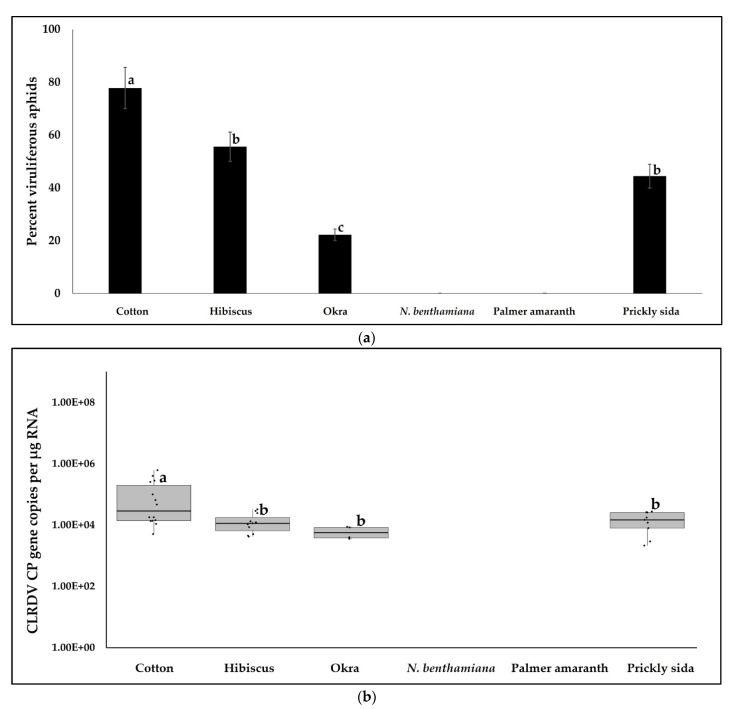
CLRDV acquisition by aphids. (**a**) Bars with standard errors represent mean percentages of aphids acquiring the virus following a 72 h AAP on infected hosts. (**b**) Boxes with whiskers represent acquisition of CLRDV CP copies by aphids following a 72 h AAP on infected hosts. The CLRDV CP copies were estimated by absolute RT-qPCR quantitation using plasmids containing the CLRDV CP gene inserts as standards. The Y-axis is represented on a logarithmic scale. Different letters on bars and boxes indicate significant differences between treatments. CLRDV incidence and accumulation were absent in *A. gossypii* following a 72 h AAP on *N. benthamiana* and Palmer amaranth.

Aphids acquired CLRDV from four hosts that were infected with CLRDV, i.e., cotton, hibiscus, okra, and prickly sida. Aphids did not survive on *N. benthamiana* plants for more than 48 h and did not test positive for the virus after a 72 h AAP on *N. benthamiana* and Palmer amaranth. The percentages of aphids acquiring virus from these four plant species were significantly different (χ^2^ = 40.659, df = 5, 107, *p* < 0.0001). The percentage of aphids acquiring CLRDV post-72 h AAP was the highest on cotton and the least on okra. The percentage of aphids acquiring CLRDV post-72 h AAP on hibiscus and prickly sida did not differ from one another (Figure 2a).

The accumulation of CLRDV in aphids following a 72 h AAP on different CLRDV-infected hosts varied significantly (*F* = 5.8419, df = 3, 21, *p* = 0.0028). The CLRDV CP copies were the highest in aphids that acquired virus from CLRDV-infected cotton than the other infected hosts. The CLRDV CP copy number did not vary between prickly sida, hibiscus, and okra (Figure 2b).

### 3.3. Back-Transmission of CLRDV to Cotton

**Figure 3:**
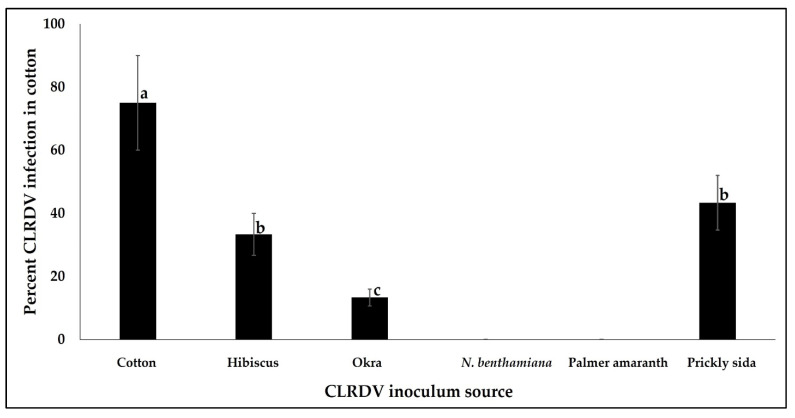
Back-transmission of CLRDV from alternate hosts to cotton. Bars with standard errors represent mean percentages of infection in cotton plants after caging 100 viruliferous aphids on the abaxial leaf surface of two-true leaf stage plants for a 72 h IAP. Inoculated plants were tested three weeks post-inoculation by RT-PCR. Aphids did not inoculate CLRDV to cotton plants following acquisition from *N. benthamiana* and Palmer amaranth. Different letters on bars indicate significant differences between treatments.

Back-transmission of virus from CLRDV-infected hosts (cotton, hibiscus, okra, prickly sida) to cotton indicated that prickly sida, hibiscus, and okra were effective inoculum CLRDV sources. As aphids did not survive on *N. benthamiana*, back-transmission from CLRDV-infected *N. benthamiana* to non-infected cotton was not feasible. Transmission of CLRDV to cotton plants varied significantly between alternate virus inoculum sources (χ^2^ = 62.485, df = 3, 177, *p* < 0.0001). The percentage of recipient cotton plants infected by CLRDV was the highest when cotton was used as the inoculum source and the least when okra was the CLRDV inoculum source. The percentage of CLRDV infection in recipient cotton plants did not vary when hibiscus and prickly sida plants were used as the CLRDV inoculum sources (Figure 3).

A subset of cotton plants that tested positive for CLRDV after back-transmission from alternate virus inoculum sources were used to assess virus accumulation by RT-qPCR. CLRDV CP copies in cotton plants did not differ significantly between alternate hosts that served as virus inoculum sources (*F* = 15.9976, df = 3, 17, *p* = 0.8987).

### 3.4. Suitability of Alternate Plant Species as Aphid Hosts

**Figure 4:**
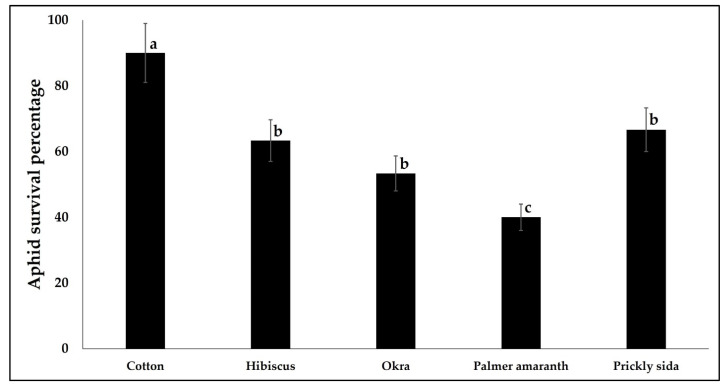
Bars with standard errors represent the mean percentage of nymphs that developed to adults on CLRDV hosts. Different letters indicate significant differences between means at α = 0.05.

The suitability of CLRDV alternate hosts for the cotton aphid was evaluated by quantifying life history traits including aphid survival, nymphal period, pre-reproductive period, reproductive period, post-reproductive period, adult period, total life span, total fecundity, and intrinsic rate of increase. The aphid survival percentage varied significantly between different hosts (χ^2^ = 17.496, df = 4, 149, *p* < 0.001). The survival (nymphs to adults) percentage was the highest on cotton and the least on Palmer amaranth. The survival percentage did not differ between hibiscus, okra, and prickly sida (Figure 4).

The median nymphal period varied significantly between alternate hosts (χ^2^ = 17.129, df = 4, *p* = 0.0018). Results were similar with reproductive period (χ^2^ = 55.312, df = 4, *p* < 0.0001), adult period (χ^2^ = 54.365, df = 4, *p* < 0.0001), and total life span (χ^2^ = 55.074, df = 4, *p* < 0.0001). The aphid nymphal period was the longest on cotton. The aphid reproductive and adult periods were similar on cotton, hibiscus, and prickly sida. The reproductive and adult periods did not differ from each other on okra and Palmer amaranth. The total life span of aphid was the longest on cotton and the shortest on okra and Palmer amaranth. The total life span did not differ between hibiscus and prickly sida. However, no significant differences in pre-reproductive and post-reproductive periods of aphids were observed on all hosts evaluated (Table 2).

**Figure 5:**
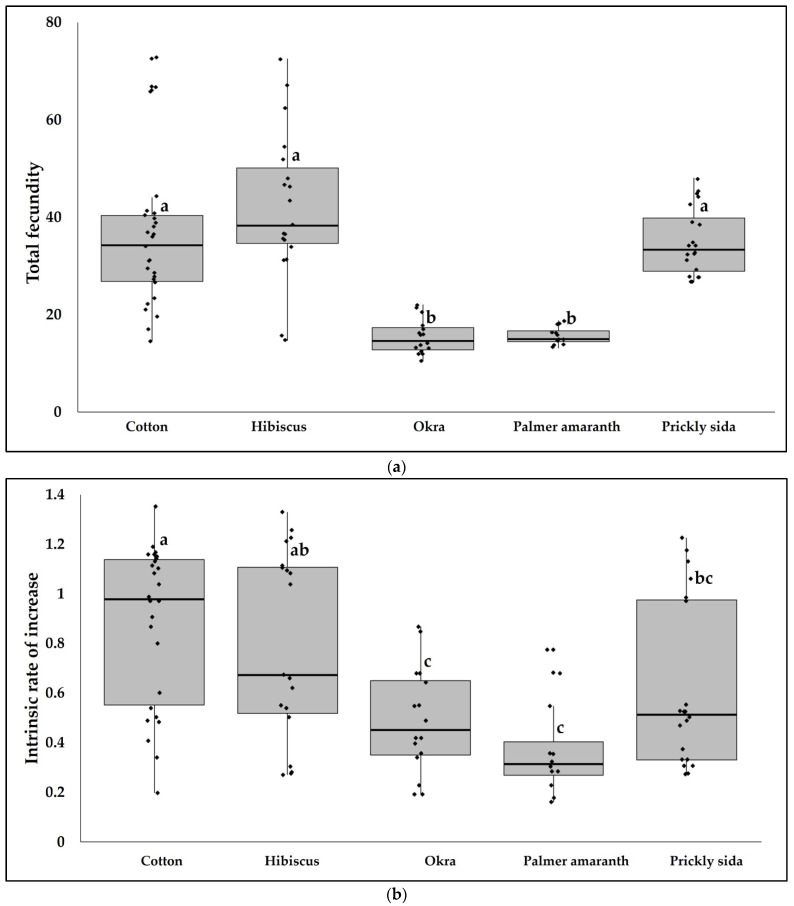
Development of *A. gossypii* on alternate hosts of CLRDV. (**a**) Boxes with whiskers represent total fecundity of aphids. (**b**) Boxes with whiskers represent intrinsic rate of increase of aphids. Different letters indicate significant differences between treatments.

Total fecundity of aphids also differed significantly between hosts (*F* = 21.7810, df = 4, 87, *p* < 0.0001). Total fecundity of aphids was lower on okra and Palmer amaranth than on cotton, hibiscus, and prickly sida. Total fecundity did not vary between cotton, hibiscus, and prickly sida (Figure 5a). The intrinsic rate of increase of aphids varied significantly among hosts (*F* = 8.4451, df = 4, 87, *p* < 0.0001) with the highest population growth on cotton (Figure 5b).

## 4. Discussion

Establishment of an introduced pathogen in an agricultural landscape depends upon several factors including the presence of alternate hosts viz., crops, ornamentals, and weeds that are annuals and/or perennials. This study evaluated several previously identified CLRDV hosts in Georgia and additional prospective hosts in the landscape. At the first stage of the investigation, 14 hosts were evaluated by aphid-mediated CLRDV inoculation. Aphid-mediated transmission assays indicated that only five of the fourteen inoculated plant species viz., hibiscus, okra, *N. benthamiana*, Palmer amaranth, and prickly sida were infected with CLRDV. CLRDV infection percentage varied up to four times between hosts indicating differential susceptibilities of alternate hosts to CLRDV. The results of this study are in agreement with other studies in the United States and South Korea that demonstrated hibiscus and Palmer amaranth as hosts of CLRDV [35]. In contrast, the results of this study are not in concordance with other earlier studies. Henbit and arrowleaf sida were not infected with CLRDV following aphid-mediated inoculation in this study, but were found to be infected with CLRDV in a field survey conducted in Georgia, USA [35]. Chickpea was not infected with CLRDV following aphid-mediated inoculation in this study, but it was found to be infected with CLRDV in India and in Uzbekistan [41,42].

Typical CLRDV symptoms such as reddening of leaves, downward curling of leaves, internodal shortening, and stunting were observed in CLRDV-infected cotton plants under field conditions but not in all instances. However, under greenhouse conditions adopted in this study, CLRDV symptoms were not observed in any of the CLRDV-infected hosts. These results suggest that CLRDV symptoms could be highly influenced by abiotic factors, which remain to be evaluated. Similarly, another study from Georgia identified asymptomatic CLRDV infection in field-grown cotton and weeds in the landscape but did not conduct any transmission assays [35]. These findings suggest that asymptomatic infections of CLRDV could also be prevalent in the landscape. Asymptomatic infection in alternate hosts does not seem to be uncommon involving viruses within *Solemoviridae*. For instance, symptomless infections also were recorded in alternate hosts of barley yellow dwarf virus (BYDV) [43].

Regardless of symptom expression, CLRDV-infected cotton plants grown in season as well as volunteers in the spring and alternate hosts can act as virus inoculum sources and influence CLRDV epidemics [35]. Two parameters could determine the ability of these hosts to function as inoculum sources: (i) accumulation of CLRDV, and (ii) CLRDV acquisition by aphids above the inoculum threshold to induce infection in inoculated plants. This study evaluated both parameters. CLRDV CP copies in hosts varied up to five orders of magnitude between CRLDV-infected hosts examined in this study. An earlier study on another polerovirus reported similar results, in which potato leafroll virus (PLRV) accumulation in alternate, host hairy nightshade (*Solanum saccharoides* L.), was lower than in its crop host potato (*Solanum tuberosum* L.) [44]. Despite the higher CLRDV accumulation in hibiscus than in cotton, a higher percentage of aphids acquired the virus from cotton than from hibiscus in this study. In another study with BYDV, aphids acquired and inoculated the virus more efficiently from an infected host with reduced virus accumulation than from an infected host that accumulated more virus [45,46]. The percentage of aphids acquiring CLRDV, and amount of CLRDV acquired by aphids on infected hibiscus, okra, and prickly sida plants was less than aphids on cotton. Differences in CLRDV aphid acquisition between hosts could be influenced by host suitability that encourages prolonged phloem feeding due to innate preferences or introduced by an experimental artefact, as aphids used in this study were reared on cotton plants for several generations. Another reason could be that virus-induced phenotype in hosts could differentially alter plant suitability to vectors. For instance, the phenotype induced by tomato yellow leaf curl virus (TYLCV) in some tomato cultivars was visually apparent than others and encouraged whitefly settling, which could have facilitated enhanced virus acquisition from those cultivars than others evaluated [36]. Similar instances of vector modulation that could enhance virus acquisition also have been reported in BYDV- and PLRV-infected hosts when compared with a host infected by non-persistently transmitted potato virus Y and mechanically transmitted potato virus X [45,47,48].

Inoculation efficiency of aphids that acquired CLRDV from cotton and alternate hosts were evaluated via back-transmission assays. Aphids were able to back-transmit CLRDV from infected hibiscus, okra, and prickly sida to non-infected cotton seedlings. The transmission efficiency of CLRDV from infected alternate hosts to cotton was lower than from infected cotton to cotton in this study. Similarly, a higher percentage of aphid-mediated transmission of BYDV was recorded from infected barley (*Hordeum vulgare* L.) to barley than from infected *Arundo donax* L. (alternate host) to barley [49]. In contrast, BYDV transmission efficiency from infected *Ventenata dubia* (Leers) Coss. (alternate host) to barley was higher than from infected barley to barley [43]. Additionally, PLRV transmission efficiency from infected hairy nightshade (alternate host) to potato was higher than from infected potato to potato [44]. Results from this study indicate that none of the alternate hosts of CLRDV identified are better inoculum sources of the virus as in the case of BYDV and PLRV.

Suitability of alternate hosts to vectors could play a significant role in their ability to support vector populations and facilitate CLRDV spread. Aphid fitness experiments conducted in this study assessed the differential ability of hosts to function as vector reservoirs. The overall survival and fitness of the cotton aphid were similar on cotton, hibiscus, and prickly sida plants in this study. In an earlier study, survival and reproduction of cotton aphids on *Hibiscus syriacus* L. were similar to aphids on cotton [50]. Additionally, the fecundity and intrinsic rate of increase were significantly lower in aphids on okra plants in this study. The reduction in survival and reproduction of *A. gossypii* on some hosts could be influenced by their intrinsic adaptability to hosts or prior exposure to certain hosts [50]. The aphid colony used in this study was reared on cotton. Host adaptability and effects on survival and reproduction in other hosts have been reported extensively for several species of aphids: *Sitobion avenae* Fabricius [51], *Acyrthosiphon pisum* Harris [52], *Aphis spiraecola* Patch [53], *Rhopalosiphum maidis* Fitch [54], and *Aphis fabae* Scopoli [55]. The host range of insect herbivores can be influenced by host nutritional quality [56,57]. Aphids with a narrower host range such as *A. gossypii* feeding on different plant species could be exposed to different nutrient profiles leading to differential host utilization [58,59]. Despite reduced fitness in hosts such as hibiscus, okra, and prickly sida in comparison with cotton, *A. gossypii* can survive, and possibly overwinter on its alternate hosts and affect CLRDV epidemics.

## 5. Conclusions

This study identified four alternate hosts that could function as inoculum sources, which mostly (except for Palmer amaranth) facilitated the back-transmission of CLRDV to cotton. Palmer amaranth is commonly present in the landscape, and it has been shown to influence epidemics of a whitefly-transmitted virus, TYLCV, in the same landscape [60]. Reduced virus accumulation and poor aphid fitness in Palmer amaranth in comparison with other alternate hosts could have prevented the back-transmission of CLRDV to cotton in this study. Hibiscus, okra, and prickly sida served as effective alternate hosts for both CLRDV and *A. gossypii* and influenced the back-transmission of CLRDV to cotton. In addition to the ability to serve as virus inoculum sources and vector reservoirs, the phenological occurrences of the alternate hosts in the presence and absence of the crop host as well as the spatial and temporal distribution of alternate hosts in relation to the crop host could critically influence virus epidemics. By that assessment, it appears that hibiscus and prickly sida could be critical players in reoccurring CLRDV epidemics. Prickly sida is typically present throughout the cotton production landscape, and it can exist in the landscape as an annual or overwinter under appropriate conditions, whereas hibiscus is typically grown as a perennial ornamental. Cotton regrowth in the spring (volunteers) also has tested positive for CLRDV [35], but cotton volunteers often are restricted to areas with warm winter temperatures. Cotton regrowth also could be common under specific production practices (strip tillage) in conjunction with mild winter [35]. It appears that both cotton and alternate hosts of CLRDV and aphids could be playing a role in establishment of CLRDV in the agricultural landscapes of the southeastern United States.

## Figures and Tables

**Table 1:** List of primers used for CLRDV detection and quantitation.

S.No.	Primer Pair	Sequence	Amplicon Size	Purpose
1	CLRDV3675F	CCACGTAGRCGCAACAGGCGT	* CP = 310 bp [5,10]	Endpoint PCR
	Pol3982R	CGAGGCCTCGGAGATGAACT		
2	CLRDV-CP5L	TGGAGGACCAGGAGCTTCAA	109 bp (This study)	qPCR
	CLRDV-CP5R	TGCCGGGCAATCTGATAAAG		
	CLRDV-CP5probe	TCTCTCGGGAAGTTCCTCAG		

* CP = Coat Protein.

**Table 2:** Developmental time (median days) of *A. gossypii* feeding on alternate hosts.

Host	N	NP	PrRP	RP	PoRP	AP	LS
Cotton	27	5 (2–7) a	1 (1–6) a	15 (6–23) a	2 (1–11) a	17 (10–35) a	23 (14–39) a
Hibiscus	19	4 (2–6) ab	2 (1–4) a	11 (2–15) a	1 (1–6) a	15 (9–20) a	19 (13–22) b
Okra	16	4 (3–7) ab	1 (1–2) a	5 (3–9) b	2 (0–3) a	8 (6–12) b	13 (9–16) c
Palmer amaranth	12	4 (2–6) ab	2 (1–3) a	6 (3–9) b	2 (0–4) a	8 (7–15) b	13 (10–18) c
Prickly sida	20	3 (2–6) b	2 (1–4) a	12 (7–17) a	2 (1–4) a	17 (11–25) a	21 (13–27) b

Different letters indicate significant differences between hosts evaluated (*p* < 0.05). N = Number of aphid nymphs monitored to adulthood, NP = nymphal period, PrRP = pre-reproductive period, RP = reproductive period, PoRP = post-reproductive period, AP = adult period, LS = total life span.

## Supplementary Materials

The following supporting information can be downloaded at: https://www.mdpi.com/article/10.3390/v14102249/s1, Section S1: Number of aphids used for CLRDV- transmission; Section S2: Plants inoculated by viruliferous and non-viruliferous aphids; Section S3: CLRDV host range; Section S4: CLRDV detection from seeds of hosts; Section S5: Relative quantitation of CLRDV P1 gene in plant hosts. Table S1: Number of aphids used for CLRDV transmission from virus-infected to non-infected cotton plants; Table S2: Number of CLRDV-inoculated plant samples; Table S3: Seeds of CLRDV hosts tested for the presence of virus; Table S4: List of primers used for CLRDV P1 and host actin genes; Figure S1: Photographs of CLRDV-inoculated and mock-inoculated plants 21 days post aphid-mediated inoculation; Figure S2: Normalized abundance of CLRDV P1 in relation with the housekeeping gene [61,62,63,64,65].
