# Supplementary material for: Prospective Alternate Hosts of an Emerging Polerovirus in Cotton Landscapes in the Southeastern United States
Source: Viruses. 2022 Oct 13;14(10):2249. doi: 10.3390/v14102249 (PMC9611507; doi:10.3390/v14102249)
Supplement: Supplementary file 1 [file viruses-14-02249-s001.zip › viruses-1920551-supplementary.pdf]

viruses

MDPI

Supplementary File

Prospective Alternate Hosts of an Emerging Polerovirus in Cot-Ton Landscapes in the Southeastern United States

Sudeep Pandey [1], Sudeep Bag [2], Phillip Roberts [3], Kassie Conner [4], Kipling S. Balkcom [5], Andrew J. Price [5], Alana L. Jacobson [6] and Rajagopalbabu Srinivasan [1,*]

[1] Department of Entomology, University of Georgia, 1109 Experiment Street, Griffin, GA 30223, USA
[2] Department of Plant Pathology, University of Georgia, 2360 Rainwater Road, Tifton, GA 31793, USA
[3] Department of Entomology, University of Georgia, 2360 Rainwater Road, Tifton, GA 31793, USA
[4] Alabama Cooperative Extension System, Auburn University, 961 S. Donahue Dr., Auburn, AL 3684, USA
[5] USDA, ARS, Soil Dynamics Research, 411 S. Donahue Dr., Auburn, AL 36832, USA
[6] Department of Entomology & Plant Pathology, Auburn University, 310 Funchess Hall, Auburn, AL 36849, USA
* Correspondence: babusri@uga.edu

S1. Number of aphids used for CLRDV-transmission

The number of aphids to facilitate optimal CLRDV transmission was evaluated. Three to five weeks post germination, five potted plants of cotton were placed in a new insect-proof cage. Adult viruliferous aphids obtained after 72h of acquisition access period (AAP) on CLRDV-infected cotton plants were caged on the abaxial side of each non-infected cotton plant using leaf cages. Aphids were provided with a 72h inoculation access period (IAP) on test plants, following which the leaf cages were removed. After the IAP, cotton plants were sprayed with imidacloprid (1% Montana 2F, Rotam, Greensboro, NC, USA) to eliminate any remaining aphids. The topmost young leaves with petiole were collected from each inoculated test plant at three weeks post inoculation and tested for the presence of CLRDV by RT-PCR.

Results indicated that consistent virus transmission was obtained while using 100 aphids. Therefore, 100 aphids were used in all future transmission experiments. CLRDV-transmission was not achieved with 10 aphids in all three repeats (Table S1).

Table S1: Number of aphids used for CLRDV transmission from virus-infected to non-infected cotton plants

	**No. of CLRDV-infected/inoculated cotton plants**			
**Repeats**	**10 aphids**	**20 aphids**	**50 aphids**	**100 aphids**
I	0/5	1/5	2/5	5/5
II	0/5	0/5	1/5	4/5
III	0/5	1/5	3/5	4/5
Total	0/15	2/15	6/15	13/15

S2. Plants inoculated by viruliferous and non-viruliferous aphids

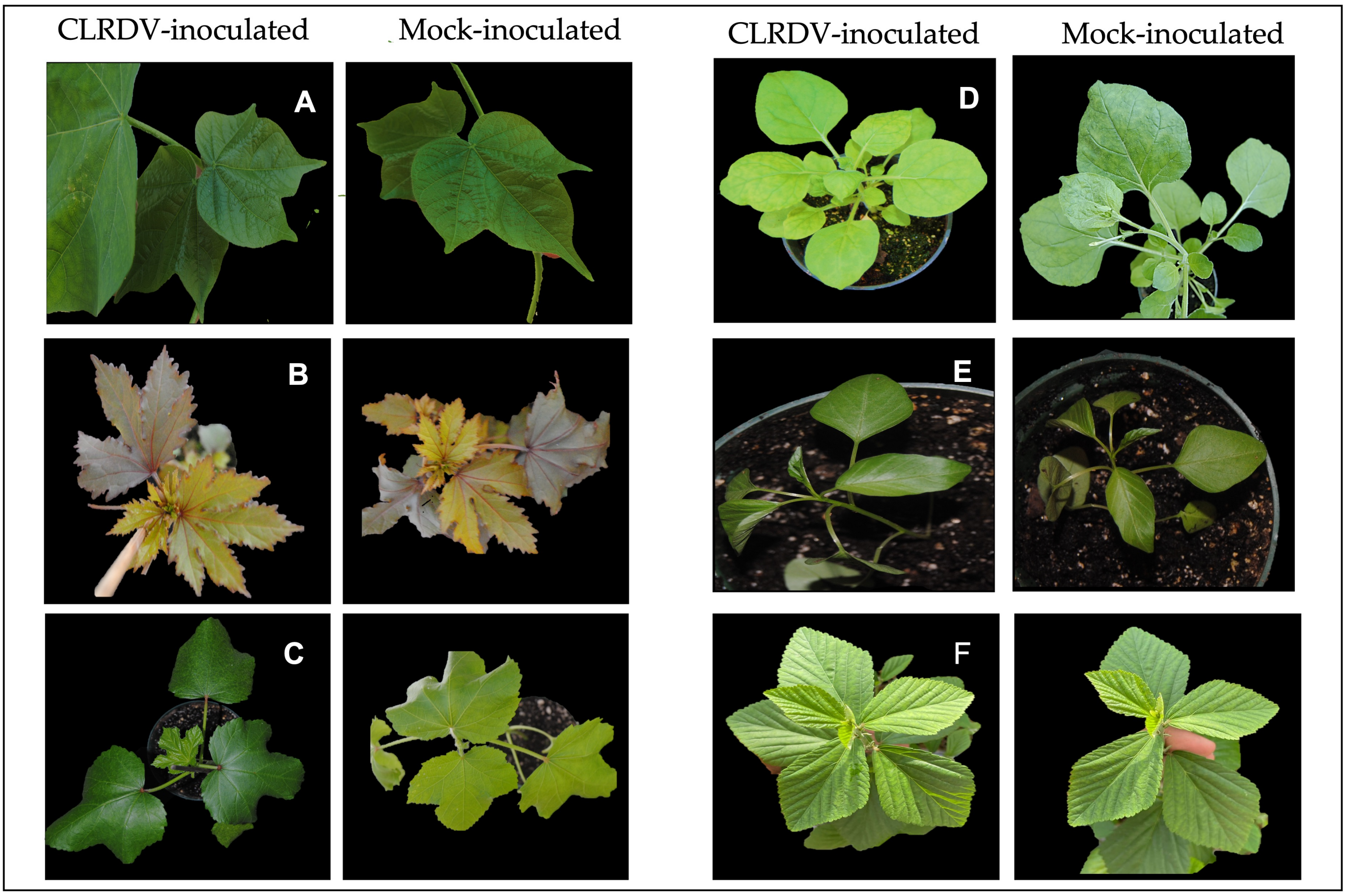

Figure S1. Photographs of CLRDV-inoculated and mock-inoculated plants 21 days post aphid-mediated inoculation. Plants inoculated with viruliferous and non-viruliferous aphids (mock-inoculated) were tested by RT-PCR. None of the mock-inoculated plants tested positive for CLRDV. A= Cotton, B= Hibiscus, C= Okra, D= *N. benthamiana,* E= Palmer amaranth, F= Prickly sida.

S3. CLRDV host range

Aphid-mediated CLRDV inoculation on 14 plant species and cotton resulted in CLRDV infection in five plant species along with cotton. None of the remaining nine plant species viz., abutilon, marshmallow, hollyhock, summer squash, country mallow, chickpea, arrowleaf sida, evening primrose, and henbit tested positive for CLRDV tested via RT-PCR (Table S2). RT-qPCR also indicated no CLRDV accumulation in these nine hosts that tested negative via RT-PCR.

Table S2: Number of CLRDV-inoculated plant samples

S.No.	Host	No. of plants CLRDV-infected/inoculated
1	Abutilon	0/20
2	Marshmallow	0/20
3	Hollyhock	0/20
4	Summer squash	0/20
5	Country mallow	0/20
6	Chickpea	0/20
7	Arrowleaf sida	0/20
8	Evening primrose	0/20
9	Henbit	0/20

S4. CLRDV detection from seeds of hosts

Seeds of hosts (cotton, hibiscus, okra, *N. benthamiana*, Palmer amaranth, and prickly sida) used in aphid-mediated transmission experiment were used for CLRDV detection. For cotton, hibiscus, and okra, individual seeds were used for RNA extraction; whereas for *N. benthamiana*, Palmer amaranth, and prickly sida, a pool of ten seeds were used for RNA extraction. RNA from the pool of ten seeds of *N. benthamiana*, Palmer amaranth, and prickly sida was considered as one sample. Seeds were soaked in water for 24h and then RNA was extracted using the Spectrum™ Plant Total RNA kit (Sigma-Aldrich, St. Louis, MO, USA) following manufacturer's instructions. The presence or absence of CLRDV infection was determined by RT-PCR as explained in section 2.3. Infected leaf tissue was used as the positive control.

Results indicated that CLRDV was not present in the seeds of CLRDV hosts (cotton, hibiscus, okra, *N. benthamiana*, Palmer amaranth, and prickly sida) (Table S3).

Table S3: Seeds of CLRDV hosts tested for presence of virus

S.No.	Host	No. of CLRDV detected / tested seed samples
1	Cotton	0/5
2	Hibiscus	0/5
3	Okra	0/5
4	*N. benthamiana*	0/5
5	Palmer amaranth	0/5
6	Prickly sida	0/5

S5. Relative quantitation of CLRDV P1 gene in plant hosts

Primers for the P1 gene was designed in house and the respective housekeeping gene (actin) from each host (except prickly sida) were obtained from the literature (Table S4). Relative quantities of CLRDV P1 gene was normalized with respective host genes. Two microliters of diluted cDNA (diluted 1:20) was used as template for the qPCR with three technical replicates. For each sample, relative quantitation was achieved by the $2^{-\Delta\Delta Ct}$ method. Relative qPCR was performed using 7.5 µl GoTaq® qPCR Master Mix (Promega, Madison, WI, USA) mixed with 0.5 µl of each primers (0.3 µM), 1µl of cDNA, and nuclease-free distilled water for a final volume of 15 µl in a QuantStudio™ 3 Real-Time PCR System (Applied Biosystems by Thermo Fisher Scientific, Waltham, MA, USA). The qPCR conditions included an initial denaturation step at 95°C for three minutes followed by 40 cycles at 95°C for 15s and 60°C for one minute. Finally, the melting curve analysis was conducted to evaluate the specificity of the fluorescence signal.

Table S4: List of primers used for CLRDV P1 and host actin genes

S.No.	Primer Pair	Sequence	Amplicon size	References
Cotton	F	TTGCAGACCGTATGAGCAAG	129bp	(Artico et al. 2010)
	R	ATCCTCCGATCCAGACACTG		
Hibiscus	F	TTGCAGACCGTATGAGCAAG	150bp	(Niu et al. 2017)
	R	ATCCTCCGATCCAGACACTG		
Okra	F	ACACTGTGCCAATCTATGAAG	145bp	(Zhang et al. 2022)
	R	ACAATTTCCCGCTCAGCAGTG		
N. benthamiana	F	TCCTGATGGGCAAGTGATTAC	114bp	(Pupim et al. 2008)
	R	TTGTATGTGGTCTCGTGGATTC		
Palmer amaranth	F	CGTGACCTGACTGATTACCTTA	178bp	(Liu et al. 2012)
	R	GCTCGTAGTTCTTCTCAATGGC		
CLRDV P1	F	CCGATAGTTCCTGAGTCGGC	189bp	This study
	R	TGCCACTTGTTTGGCTACCT		

The results indicated that CLRDV P1 gene level in hosts varied significantly (F = 28.634, df = 4, 22, P < 0.0001). CLRDV P1 gene levels were the highest in *N. benthamiana* and the lowest in Palmer amaranth and okra. CLRDV P1 gene levels in cotton and hibiscus did not differ from each other (Figure. S2). The results were similar to CLRDV CP copies in hosts as described in section (3.1).

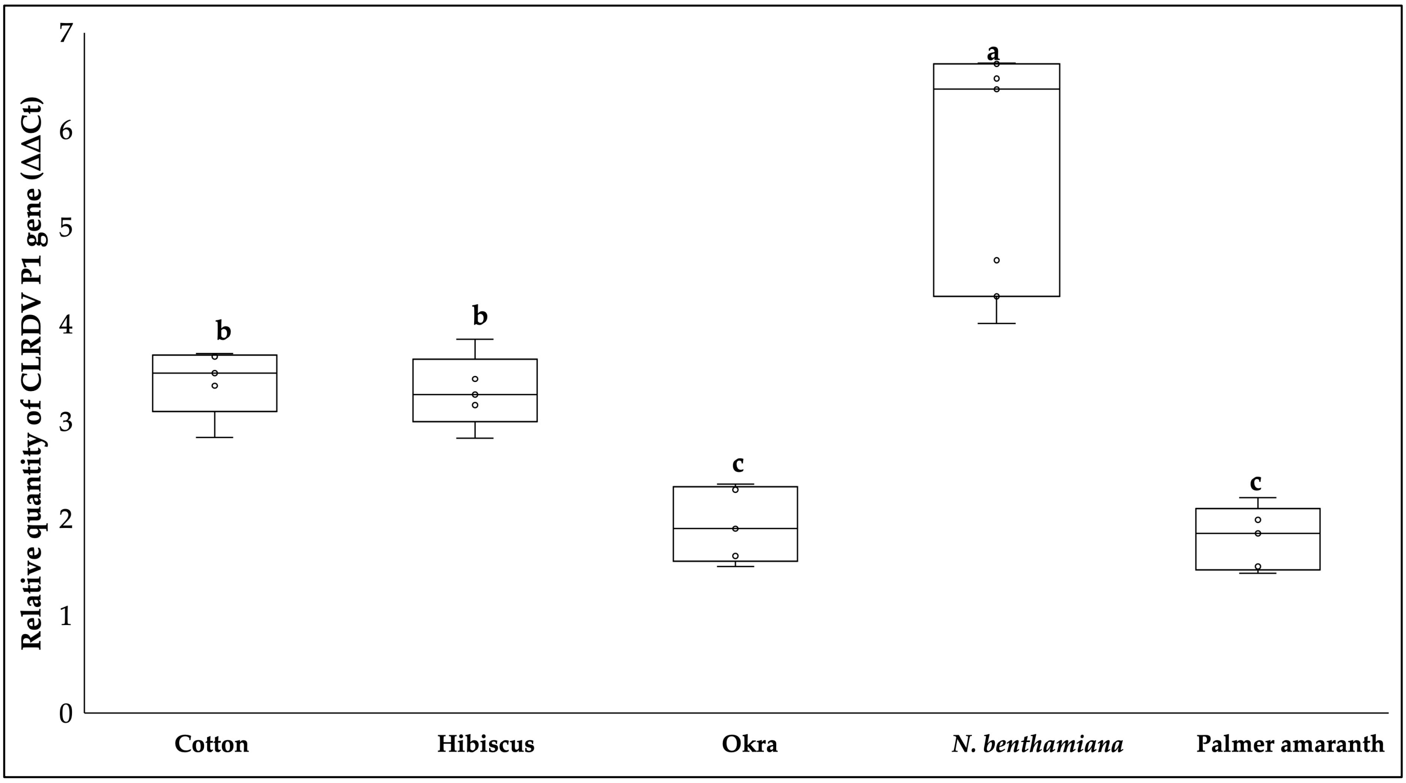

Figure S2. Normalized abundance of CLRDV P1 in relation with the housekeeping gene. Boxes with whiskers represent CLRDV accumulation on infected hosts that tested positive for CLRDV three weeks post aphid-mediated inoculation. Different letters on boxes indicate significant differences between treatments.

References:

Artico, S., Nardeli, S. M., Brilhante, O., Grossi-de-Sa, M. F., and Alves-Ferreira, M. 2010. Identification and evaluation of new reference genes in Gossypium hirsutum for accurate normalization of real-time quantitative RT-PCR data. BMC Plant Biol. 10:49.

Liu, D., Shi, L., Han, C., Yu, J., Li, D., and Zhang, Y. 2012. Validation of Reference Genes for Gene Expression Studies in Virus-Infected Nicotiana benthamiana Using Quantitative Real-Time PCR. PLoS One. 7.

Niu, X., Chen, M., Huang, X., Chen, H., Tao, A., Xu, J., et al. 2017. Reference gene selection for qRT-PCR normalization analysis in kenaf (Hibiscus cannabinus L.) under abiotic stress and hormonal stimuli. Front. Plant Sci. 8:771.

Pupim, O., Schuster, I., Pinto, R. B., Pires, E., Belot, J. L., Silvie, P., et al. 2008. Inheritance of resistance to cotton blue disease. Pesqui. Agropecu. Bras. 43:661–665.

Zhang, J. R., Feng, Y. Y., Yang, M. J., Xiao, Y., Liu, Y. S., Yuan, Y., et al. 2022. Systematic screening and validation of reliable reference genes for qRT-PCR analysis in Okra (Abelmoschus esculentus L.). Sci. Reports 2022 121. 12:1–10.
